# Capable and credible? Challenging nutrition science

**DOI:** 10.1007/s00394-017-1507-y

**Published:** 2017-07-17

**Authors:** Bart Penders, Anna Wolters, Edith F. Feskens, Fred Brouns, Machteld Huber, Els L. M. Maeckelberghe, Gerjan Navis, Theo Ockhuizen, Jogchum Plat, Jan Sikkema, Marianne Stasse-Wolthuis, Pieter van ‘t Veer, Marcel Verweij, Jan de Vries

**Affiliations:** 1 Department of Health, Ethics and Society, Care and Public Health Research Institute (CAPHRI), Maastricht University, Maastricht, The Netherlands; 2 Division of Human Nutrition, Wageningen University, Wageningen, The Netherlands; 3 Department of Human Biology and Movement Sciences, Nutrition and Translational Research in Metabolism (NUTRIM), Maastricht University, Maastricht, The Netherlands; 4 Institute for Positive Health, Amersfoort, The Netherlands; 5 Institute for Medical Education, University of Groningen, University Medical Center Groningen, Groningen, The Netherlands; 6 Department of Nephrology, University of Groningen, University Medical Center Groningen, Groningen, The Netherlands; 7 Nutricom Consultancy, Rumpt, The Netherlands; 8 Center for Development and Innovation, University of Groningen, University Medical Center Groningen, Groningen, The Netherlands; 9 Section Communication, Philosophy and Technology, Department of Social Sciences, Wageningen University, Wageningen, The Netherlands; 10 De Vries Nutrition Solutions, Gorssel, The Netherlands

**Keywords:** Nutrition science, Credibility, Capability, Inclusiveness, Evidence, Real-world experiments

## Abstract

Nutrition science has enriched our understanding of how to stay healthy by producing valuable knowledge about the interaction of nutrients, food, and the human body. Nutrition science also has raised societal awareness about the links between food consumption and well-being, and provided the basis for food regulations and dietary guidelines. Its collaborative and interdisciplinary research has accomplished much, scientifically and socially. Despite this, nutrition science appears to be in crisis and is currently confronted with a public reluctance to trust nutritional insights. Though deflating trust is a general phenomenon surrounding the scientific community, its impact on nutrition science is particularly strong because of the crucial role of nutrition in everyone’s daily life. We, a Dutch collective of nutritionists, medical doctors, philosophers and sociologists of science (http://www.nutritionintransition.nl), have diagnosed that nutrition science is meeting inherent boundaries. This hampers conceptual and methodological progress and the translation of novel insights into societal benefit and trust. In other words, nutrition science is facing limitations to its capability and credibility, impeding its societal value. We take up the challenge to halt the threatening erosion of nutrition science’s capability and credibility, and explore a way forward. We analyse limitations to capability and credibility, then argue that nutrition science is caught in a vicious circle, and end by offering some suggestions to transcend the limitations and escape the current deadlock. We invite nutritional experts as well as scholars from adjacent disciplines to engage in the discussion.

## Introduction

Nutrition science has enriched our understanding of how to stay healthy by producing valuable knowledge about the interaction of nutrients, food, and the human body. Nutrition science also has raised societal awareness about the links between food consumption and well-being, and provided the basis for food regulations and dietary guidelines [1, 2]. Its collaborative and interdisciplinary research has accomplished much, scientifically and socially. Despite this, nutrition science appears to be in crisis and is currently confronted with a public reluctance to trust nutritional insights. Though deflating trust is a general phenomenon surrounding the scientific community, its impact on nutrition science is particularly strong because of the crucial role of nutrition in everyone’s daily life [3].

We, a Dutch collective of nutritionists, medical doctors, philosophers and sociologists of science (http://www.nutritionintransition.nl), have diagnosed that nutrition science is meeting inherent boundaries. This hampers conceptual and methodological progress and the translation of novel insights into societal benefit and trust. In other words, nutrition science is facing limitations to its capability and credibility, impeding its societal value.

Ours is not the first critical assessment of state of the (inter)discipline [4, 5]. Our analysis resonates with expressed concerns in the literature about replicability and real-life relevance [6–8] and anchors these concerns in debates about the added value of the sciences more in general [9] and nutrition science in particular [10, 11]. We take up the challenge to halt the threatening erosion of nutrition science’s capability and credibility, and explore a way forward. In the following two sections, we analyse limitations to capability and credibility, then argue that nutrition science is caught in a vicious circle, and end by offering some suggestions to transcend the limitations and escape the current deadlock. We invite nutritional experts as well as scholars from adjacent disciplines to engage in the discussion.

## Capability limits

Our first thesis is that the bulk of knowledge that is currently flowing from nutritional research institutes does not match the major societal challenges of the twenty-first century, i.e. the demographic transition towards an ageing population, the increasing burden of non-communicable disease attributable to lifestyle, and the urgent needed for sustainability. The mismatch imposes limits to the *capability* of nutrition science to contribute to real-world health. This capability is restricted in at least three ways: by the questions we pursue, by the technical and methodological characteristics of our approach, and by the organisation of nutrition science.

The nutrition *questions* have evolved throughout the centuries. The alleviation of nutritional deficiencies and the discovery of vitamins were followed by the heyday of nutrition science as applied biochemistry. Presently, mirroring the clinical evolution towards evidence-based medicine, the quest is for evidence-based nutrition, which underpins guidelines, health claims and policies. Yet the questions for the next decennia in the context of (regional) nutrition abundance are very different. New challenges lie in gaining healthy life years, preventing multifactorial diseases and multi-morbidity, designing personalised and public health nutrition strategies, providing healthy and safe diets, but also in realising food and nutrition security, and in working on a sustainable food system.

Hence, the *methods* in nutrition science need to change to accommodate these new questions. Reductionism is indispensable to answer questions related to specific ingredients and has been a highly successful approach for nutrition science for decades [1, 2]. However, and possibly as a result of this, exclusive emphasis on thinking in terms of substances easily becomes a dogma [12], hampering nutrition science’s ability to diversify its views on individual and public health beyond the statistical or biochemical behaviour of single molecules. To investigate the effects of isolated substances and to demonstrate causality as required by the reductionist approach, the randomised controlled trial (RCT) is the highest ranked tool in the evidence pyramid. However, in nutrition, it is difficult to transfer such trial outcomes to diets and food patterns in daily life. The composition of foods differs according to region and climate, while dietary habits and meal patterns shift per week, month, season, and food availability. Questions elicited by this real-life picture cannot be explored in the artificial environment of the RCT. Hence, while recognising the emphasis on internal validity of RCTs, the external validity of such controlled trial results is a matter of scientific and societal concern. Nutrition science needs to actively seek and embrace the addition of new, innovative concepts to adequately study the effects of nutrition on health maintenance and disease prevention in real life, in collaboration with other relevant disciplines.

The *organisation* of nutrition science is still strongly influenced by a reductionist focus that orients public and commercial incentives in specific directions and obscures others. Partly due to changing governmental research policies, significant funding comes from the food industry. The industry is more focused on products and nutrients than on diets and food patterns, which is further strengthened by the subsequent emphasis on health claims [12]. To re-establish its capability, nutrition science needs to adapt to changing societal contexts and revisit its organisation and financial structures. It is also important to allow for novel concepts, study designs and challenging end points, such as biomarkers for maintaining health or enhancing resilience. Drawing from the interdisciplinary richness of nutrition science, alternative perspectives on health are already available, including new dynamic concepts of health [13].

## Credibility limits

Our second thesis is that the credibility of the discipline is at stake. The new US Guidelines, for example, have been attacked and the authority of organisations like the Institute of Medicine (IOM) is questioned [14]. This is in line with a general decrease in trust in institutions such as politics and science. How to diagnose this problem? In our view, credibility in science results from reciprocal communication of scientists and the public on both its (1) relevance and (2) moral character and reliability [15].

The *relevance* of nutrition science primarily consists in the increased knowledge about the long-term impact of nutrients, foods and food patterns on health maintenance and disease onset. The benefits of this knowledge at the individual and group level are not immediately obvious for the public. Few individuals will really perceive and experience the benefits of choosing their food according to the state of the art of nutrition science. What grasps the public eye are often oversimplified statements about what is or is not healthy. Yet such absolute claims, which may also originate from nutritionists, are often contested later on. These results in confusion among lay persons about what they can and cannot ‘believe’. More nuanced or not readily applicable knowledge from nutrition scientists, if communicable and communicated at all, is not often well perceived. And yet, the general public is hugely interested in food matters, witness the steady stream of diets, culinary books, cooking programs, and nutrition theories from self-appointed experts [3].

Second, the *moral character and reliability* of nutrition science and its champions seems tarnished. Competing claims, fuzzy results, interestedness, and messiness are all part of ‘normal science’, and ask for critical debate. In nutrition science that complex picture is even more intricate. Public funding being often very limited or even absent, the discipline nowadays is largely dependent on corporate money to do research at all. This begs the question of conflicts of interest and severely influences the perceived reliability of the results [16–18]. Despite the overall integrity of nutrition scientists, to the general public these public–private collaborations engender doubts on the independence and reliability of scientists.

## Vicious circle

The type of evidence we seek as nutrition scientists, the questions we ask, and the way nutrition science is funded and organised, all threaten the credibility of our discipline. To some extent, these threats, reinforced by doubts about the discipline’s relevance, integrity, and reliability, may push nutrition science to emphasise the need for more exact science, and thus downplay the role of public health and social sciences in nutrition. This effect is amplified by research institutes wishing to score with high profile, high impact publications and with ‘simple’ messages that attract media attention. This only reinforces the very reductionist paradigm that we should seek to overcome. In other words, threats to credibility may in turn threaten capability, and vice versa.

## Capable and credible

Breaking free from this vicious cycle will require different ways of organising and doing research. The pursuit of a truly capable and credible nutrition science requires *reciprocity* in the articulation of relevance and in communication and *inclusiveness* through inviting other disciplines to become co-creators of the new nutrition science. We can reach out to non-academics, ranging from breeders to patient and consumer organisations, as legitimate research collaborators. Reciprocal and inclusive research carries consequences for how we design that research, and for how we translate its results for the benefit of society.

For research design, they require a different *organisation* of research allowing this greater number of voices to co-design research, including new types of more flexible trial design (such as quasi-experimental studies and *n*-of-1 trials [6, 19]), ranging from existing strategies such as intervention mapping to more experimental participatory intervention designs [20]. For translation, the *rhetoric* of nutrition science requires adjustment, debunking myths like easy and quick weight-loss, as well as departing from the myth of pure, neutral science, able to achieve objective truth [21].

The practices we propose deviate significantly from dominant knowledge production strategies: less emphasis on controlled conditions, and few RCT-like elements. Departing from an RCT-dominant perspective entails continuous reflections on evidence (type, amount and origin), significance and validity in general, and what evidences and support allow claims of correlation or causation in particular. Instead we propose to focus on real eating practices, explicate health values of participants, and engage participants in articulating their values as well as common health outcomes [22]. Accordingly, the reinvention of nutrition science is a real-world experiment in which traditional nutritional experts share their spot at the helm [23].

Capability and credibility, drawn from the pursuit of reciprocity, inclusiveness and a humble rhetoric in research practice and research translation alike will allow us to tell compelling narratives about how nutrition science helps to gain a better understanding of the interaction of dietary habits, foods, quality of life, and health.
